# Mechanosurveillance: Tiptoeing T Cells

**DOI:** 10.3389/fimmu.2022.886328

**Published:** 2022-05-26

**Authors:** Janett Göhring, Lukas Schrangl, Gerhard J. Schütz, Johannes B. Huppa

**Affiliations:** ^1^Institute for Hygiene and Applied Immunology, Center for Pathophysiology, Infectiology and Immunology, Medical University of Vienna, Vienna, Austria; ^2^Institute of Applied Physics, TU Wien, Vienna, Austria

**Keywords:** immune surveillance, mechanical force, T-cell antigen recognition, glycocalyx, physical barriers, microvilli, membrane ultrastructure

## Abstract

Efficient scanning of tissue that T cells encounter during their migratory life is pivotal to protective adaptive immunity. In fact, T cells can detect even a single antigenic peptide/MHC complex (pMHC) among thousands of structurally similar yet non-stimulatory endogenous pMHCs on the surface of antigen-presenting cells (APCs) or target cells. Of note, the glycocalyx of target cells, being composed of proteoglycans and bulky proteins, is bound to affect and even modulate antigen recognition by posing as a physical barrier. T cell-resident microvilli are actin-rich membrane protrusions that puncture through such barriers and thereby actively place the considerably smaller T-cell antigen receptors (TCRs) in close enough proximity to APC-presented pMHCs so that productive interactions may occur efficiently yet under force. We here review our current understanding of how the plasticity of T-cell microvilli and physicochemical properties of the glycocalyx may affect early events in T-cell activation. We assess insights gained from studies on T-cell plasma membrane ultrastructure and provide an update on current efforts to integrate biophysical aspects such as the amplitude and directionality of TCR-imposed mechanical forces and the distribution and lateral mobility of plasma membrane-resident signaling molecules into a more comprehensive view on sensitized T-cell antigen recognition.

## Introduction

The adaptive immune response is an extraordinarily complex process involving a multitude of different cell types, transmitters, and effector molecules performing their intended function in diverse tissue environments, some of which are altered by disease and infection. Slight deviations within the involved mechanisms can lead to severe medical complications, namely, allergies, autoimmune diseases, hypo- or hyper-reactions to invading pathogens, and the development of cancer. The degree of fine-tuning required for immune protection becomes apparent at multiple regulatory levels controlling T-cell activation. The pivotal event preceding many of the ensuing cellular interactions concerns the specific molecular recognition of processed pathogenic peptides displayed in the context of the major histocompatibility complex (MHC and peptide-presenting MHC; pMHC) by T cells *via* their clonotypic and genetically recombined T-cell antigen receptors (TCRs).

Interactions between stimulatory pMHCs and TCRs are highly specific and confer exquisite T-cell antigen sensitivity, a *sine qua non* considering the consequences of recognition failure. It is, however, not yet clear how such a level of specificity and sensitivity is maintained, since measured biochemical affinities between TCRs and pMHCs are astonishingly low. A number of models have been conceived and tested in the last two decades. Experimental efforts focused on revealing the molecular machinery involved in antigen recognition events and led to the formulation of concepts implicating kinetic segregation, kinetic proof-reading, co-receptor involvement, ligation-triggered conformational changes, serial engagement, and the contribution of mechanical forces in early recognition events [reviewed in (1)]. These models have their merits, and the ground truth will very likely be a combination of their aspects.

**Figure 1:**
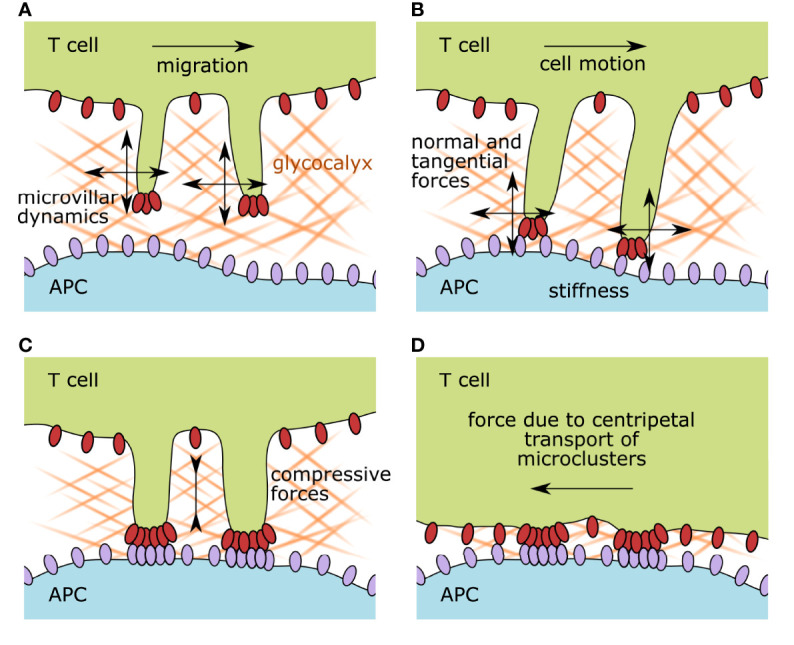
Illustration of the membrane organization of scanning and activated T cells and the accompanying possible mechanical forces affecting surface receptors. **(A)** During immune surveillance, T cells scan target cells via microvillar protrusions. The first physical barrier they encounter is the glycocalyx of the target cell. Antigen scanning speed is impacted by the glycocalyx physicochemical properties such as stiffness, density, and matrix composition, but also by the migrational speed of the T cell itself and its microvillar dynamics. **(B)** As soon as surface receptors on the microvillar tips interact with their ligands on the opposing membrane, the formed bonds experience a force vector with normal and tangential components. The surface stiffness of the target cell and the microvillar elasticity also influence the interacting receptor–ligand pairs. **(C)** Upon recognition of a cognate antigen, T-cell activation starts. Surface receptor molecules build signaling platforms while the two participating plasma membranes approach each other, compressing the remaining glycocalyx components. **(D)** After the initiation of TCR signaling, the zonal organization of the immunological synapse is established and signaling foci, called microclusters, are pulled by the actin cytoskeleton toward the center of the cell. This dragging motion is also causing mechanical strain on the involved receptor–ligand pairs. APC, antigen-presenting cell.

The complex interaction of naïve antigen-inexperienced T cells with antigen-presenting cells such as dendritic and B cells can be described on different spatial and temporal scales (see **Figure 1** for an illustration of these processes). Highly dynamic cellular interactions involving massive cytoskeletal rearrangements transpire during the entirety of the cell–cell contact. Before antigen recognition, pre-existing membrane protrusions such as microvilli scan the surface of the target cell in search of their cognate antigen (2–5). As a result, physical barriers such as the glycocalyx of the target cell are overcome by the surveilling microvilli. During scanning, surface receptors at the tip of the microvilli are subject to a large range of mechanical forces such as tensile, compressive, and shear stresses (6). Once a specific recognition event is established, the T cell receives a movement arrest signal and the two interacting cells start reorganizing their conjugation plane, which involves massive membrane undulations and the formation of other membrane projections such as invadosome-like structures (2, 7–9). Proteins within the entire conjugation plane become spatially reorganized, forming the immunological synapse with a central and peripheral domain (10).

The unique two- and three-dimensional properties of immune synapses are likely to influence the dynamics of intrinsic receptor–ligand interactions (11). For example, massive membrane rearrangements ensue after initial contact and produce spatial constraints that eventually result in the molecular segregation of membrane receptors and ligands based on the size of their extracellular domains (12). Signal-maintaining microclusters are formed, containing ligated TCRs, which are subsequently dragged by the cytoskeleton toward the center of the synapse (13–17). This active process creates drag on other constituents, causing membrane undulations due to elastic deformation and relaxation (18). In this fashion, measurable pulling forces are exerted by the regulated, centripetal flow of the cortical actin cytoskeleton (19).

Considering the temporal flow of events, one must consider that signaling events during T-cell activation are divided into early recognition events, which eventually lead to motility arrest, and signal-maintaining events. Early recognition events fulfill the purpose of fast and efficient antigen screening (20, 21), whereas later events are needed to maintain a steady T-cell response at a continuous antigenic stimulus (14, 17, 22, 23).

In this review, we highlight the cell biological and biophysical features of T-cell microvilli, which act as antigen-sensing units and, in turn, reevaluate the impact of physical barriers and mechanical forces on immune surveillance.

## Antigen Scanning Entities: Microvilli of T Cells

T cells are exceptionally motile as they roam a multitude of different environments during their life cycle (24, 25). After differentiation and selection in the thymus, naïve T cells move into the blood and lymphatic system to reach secondary lymphoid systems. In doing so, T cells are exposed to strong shear forces caused, for example, by the dynamics of the blood flow. To leave the blood stream, T cells perform a complex maneuver along the endothelial wall, comprising a selectin-mediated rolling and adhesion cycle that eventually leads to diapedesis. After entering a lymph node or tissue of interest, T cells start migrating through dense three-dimensional extracellular matrices while continuously screening antigen-presenting cells (APCs) or target cells, which they continuously encounter for cognate antigen. Upon stimulation, T cells switch their migration mode and form cell–cell interfaces, which, depending on tissue properties, are termed immunological kinapses or synapses. Subsequently, they start proliferating, and eventually start their surveilling migration anew throughout the body. Further triggering of an antigen-experienced T cell leads to the fulfillment of its effector function according to the subtype of the T cell.

During immune surveillance, the membrane ultrastructure of scanning T cells is especially dynamic in order to guarantee adaptation to different physical environments and functions. Prominent structures are the actin-rich membrane protrusions called microvilli, which play a central role in antigen surveillance.

### Physical Barriers in Immune Surveillance

An interesting aspect of T-cell interactions concerns the biophysical environment T cells experience while they are scanning their surroundings. T cells are actively probing for pathogen-derived or otherwise atypical or non-endogenous peptides presented on the surface of cells that they encounter in their migratory life. However, cells are protected by the glycocalyx, a dense and wide coat of extracellular polysaccharides and proteins, which creates a physical barrier, preventing the close apposition of the cellular membranes and, consequently, any intercellular ligand–receptor interaction (26–28). To deal with this, T cells feature membrane protrusions that can puncture through the cell-resident glycocalyx to efficiently scan large portions of a large variety of cells and drive TCR-specific signaling (2, 4, 7, 29). The glycocalyx is a naturally occurring physical barrier made of extracellular branched carbohydrates, glycolipids, glycoproteins, and proteoglycans that covers the plasma membrane of cells (30). The involved carbohydrates (i) can be directly linked to their respective anchor molecules *via* N- or O-glycosidic bonds or are independent entities within the matrix, (ii) are continuously remodeled by cellular enzymes, (iii) are primarily negatively charged, and (iv) play an important role in cellular processes such as signal amplification, adhesion, migration, and cell death (31). The complexity and height of this dense, gel-like coating can vary from 250 to >500 nm (32) and it is filled with ions, growth factors, chemo- and cytokines.

The exact composition of the glycocalyx has been characterized in detail for endothelial cells: prominent membrane-anchored proteoglycan groups are syndecans and glypicans, whereas other proteoglycans such as mimecan, perlecan, and biglycan are actively secreted into the extracellular space and blood stream (33). These proteoglycans form a dense network by associating with glycosaminoglycans (GAGs) such as heparan sulfate, dermatan sulfate, chondroitin sulfate, and hyaluronic acid, to name a few ubiquitous GAGs. Apart from these components, a large variety of glycoproteins such as selectins, integrins, and immunoglobulin-like proteins also participate in forming a dense extracellular network (33). It is beyond the scope of this review to discuss all the participating glycocalyx players, but it is important to note the existence of a complex, multi-layered, and highly dynamic layer beyond the plasma membrane that impacts any cell–cell interaction.

The glycocalyx of the T cell consists mainly of a few prominent surface proteins with large extracellular domains, namely CD43 and CD44, and the protein tyrosine phosphatases CD45 and CD148. These proteins' extracellular domains are 3–4 fold larger in length than the physiological intermembrane distance, allowing for a pMHC–TCR bond (~15 nm) (26, 34), and resemble stiff rod-like structures that are unlikely to bend in response to external tensile forces (35). Similarly, CD148 and CD45 also carry large extracellular domains but fulfill additional signaling functions in the wake of TCR triggering. During the immune synapse formation, these proteins and integrin LFA-1 binding to ICAM-1 are positioned in distinct zones creating varying intermembrane distances. This zonal reorganization after TCR triggering leads to steep membrane curvatures accompanied by dynamic membrane tension profiles in the contacting APC and T cell membranes [reviewed in (34)]. Not much is yet known with regard to the presence of typical membrane-anchored and secreted proteoglycans and glycoproteins in the glycocalyx of T cells, yet one study investigated the upregulation of Mucin-1 (a very large glycocalyx-building proteoglycan) after mitogenic stimulus in activated T cells (36) and another study detected hyaluronan (a prominent GAG) on the surface of T cells in certain conditions (37). However, given their migratory lifestyle and the necessity to visit and scan a large variety of tissues, the presence of a bulky glycocalyx network seems disadvantageous and may only be upregulated in certain scenarios such as trafficking and homing. Also, T cells exhibit a stiffness modulus of only ~85 Pa, which is very soft in comparison to other cell systems (38). This has implications for cell–cell interactions, as the cell with the lower rigidity spreads at the interface. Recent rheological measurements of the viscoelastic properties of T cells during activation show a 2–3 fold increase in stiffness after stimulation with an activating microbead (39). The authors also performed AFM experiments with T cell–APC conjugates and concluded that mechanical changes occur within seconds of initial contact (39).

The glycocalic layers of professional APCs have so far been investigated to a much lesser extent, and given the migratory life of these cells, it is unclear if the glycocalyx layers are up or downregulated in their immature/mature states or the varying lymphoid environments they reside in. One study showed the presence of Mucin-1 on dendritic cells *in vivo* (40). Also, a recent publication investigated the contribution of hyaluronan (HA) to the glycocalyx layer of migratory DCs and discovered a 400–500 nm thick glycocalyx layer anchored and regulated by the HA-receptor CD44 (41). The study also showed that the HA content within the glycocalyx was upregulated for mature DCs compared to immature DCs, and consequently, that the presence of HA glycocalyx is necessary for trafficking over the lymphatic endothelium and allowing crawling along the endothelia (41, 42). This aspect becomes crucial considering that long-lived MHC-dependent T cell–DC interactions occur on the luminal side of afferent lymphatic capillaries (43, 44).

An interesting mechanism was recently uncovered in the work of Imbert et al., characterizing the immune-modulatory impact of the glycocalyx on the target cell during phagocytosis in *in vitro* and *in vivo* settings (45). The elegant experiments showed that the presence of a repulsive glycocalyx on target cells prevents phagocytosis, leading to effective immune evasion, and that, similarly the upregulation of glycocalyx layers on the phagocyte itself led to the same inhibition of phagocytosis. The study clearly showed that glycocalyx layers can actively prevent immune recognition and the triggering of surface receptors by restricting their accessibility. Along this line of thought, cancerous cells have been shown to alter their glycocalyx composition (46, 47), in that they vary its height and other biophysical parameters, thereby promoting their immune evasion capabilities (48, 49).

Biophysical parameters of the glycocalyx have been measured using AFM nano-indentation (50), resulting in an elastic modulus of around 0.39 kPa. The plasma membrane lacking a glycocalyx has been found to be less flexible with an elastic modulus of about 3 kPa. The corresponding stiffness of cells has been experimentally determined to be in the range of 10 Pa to 10 kPa, the variability resulting from different cell types and confounding parameters like intracellular pressure and actin–myosin contractility of the underlying cytoskeleton and differences in methodology (51). Interestingly, spread mesenchymal stem cells exhibit a larger stiffness modulus than rounded ones, with 3.2 and 2.5 kPa, respectively (52). The stiffness of monocyte-derived dendritic cells has been reported to be in the range of 0.5 kPa, with their stiffness changing upon inflammation (38), whereas another study reports a 2–3 fold increase in stiffness for maturing DCs (within the range of 2 to 8 kPa) (53).

All these aspects become relevant when considering the sensitivity of the T cell toward different substrate stiffnesses. The stiffness and porosity of the glycocalyx of both T cell and APC may hence be critical for antigen accessibility, whereas the APC cortex stiffness may be critical for antigen sensitivity and subsequent signaling (54). It has been comprehensively shown that the stiffness of the ligand-presenting surface impacts T cell signaling, proliferation, and differentiation (54–59). Some of these studies report a positive correlation between the substrate stiffness and T-cell activation (55–57), while others show an inverse correlation (54, 58). The investigated stiffness ranged from a few Pa to MPa, while most studies report a maximum cellular response at a substrate stiffness of 100 kPa. In a more physiological setting, Blumenthal et al. investigated the impact of the cortical stiffness of dendritic cells on the T-cell response (53): by varying the stiffness of stimulatory hydrogels in the physiological range of immature and mature DCs (2 to 8 kPa, respectively), the authors showed an increase in CD4^+^ T-cell antigen sensitivity and responses under stiffness conditions reflecting that of mature DCs. Strikingly and in contrast, CD8^+^ T cells only showed modest sensitivity toward stiffness. By substituting pMHC as a stimulatory ligand for activating antibodies, the authors revealed a dependence of the stiffness response on the type of TCR–ligand engagement. Within the measured physiological stiffness range (2–8 kPa), this limitation dramatically affected T-cell responsiveness, as only treatment with pMHCs but not with antibodies triggered T-cell activation under these conditions, except for very high ligand densities (53). Considering the effect of substrate stiffness on T-cell effector function, recent studies reporting on the vulnerability of cancer cells to T-cell cytolytic activity showed a positive correlation between immune response and cellular rigidity (60). As a consequence, cancer cells may actively evade antitumor immune responses by softening their cortical actin cytoskeleton.

### Microvilli Dimensions and Localization

In the mid-1970s to 1980s, efforts were made to morphologically characterize lymphocytes using the then newly developed technique of scanning electron microscopy, and consequently, microvillar protrusions were discovered covering the surface of lymphocytes (61–63). These membrane structures were described as being dynamic and dependent on the cell cycle, temperature, inter-cell contact, and even antigenic stimulus (64). Twenty-five years later, it was possible to detect the presence of membrane protrusions on the surface of circulating T cells (65), and prior to engagement with antigen-presenting cells (2, 4, 66) and within lymph nodes (3). These studies demonstrated the existence of such membrane protrusions during initial contact formation. Cai et al. quantified the occurrence of microvillar protrusions *via* Lattice Light Sheet Microscopy: the cell membrane is densely packed with highly dynamic microvillar protrusions that cover 98% of the cellular surface over a time period of 1 min (2). After the formation of the immunological synapse, the membrane structure ultimately becomes more planar, creating wider close-contact zones between the participating cell membranes (66, 67). Leukocyte microvillar protrusions, used for initial tethering to and rolling along the endothelia within the high shear force conditions of the blood stream (9, 68, 69), will not be the focus of this review in view of their largely different functions and surface receptor composition.

Electron-microscopic snapshots of T cells interacting with antigen-presenting cells allow the characterization of the morphology of membrane protrusions on resting, scanning, and activating T cells. The protrusions are 300–400 nm long (median, up to 4 µm has been reported) and 70–350 nm in diameter with a density of 3–4 protrusions per µm^2^ on a resting T cell (3, 5, 7, 65, 70). So far, we are not aware of any comparative study quantifying the abundance of microvilli on circulating and scanning T cells. Interestingly, the dimensions of microvillar protrusions are similar when comparing murine and human blood-isolated lymphocytes, even though human lymphocytes are in general twice as large in cell diameter (65). This conserved feature size of the microvilli indicates the existence of a common physical parameter T cells must overcome during their life time, e.g., the thickness of the glycocalyx during immune surveillance or similar shear forces during rolling tethering in the blood stream. Upon contact formation with a professional antigen-presenting cell, the protrusion tips form close contacts with the opposing cellular membrane in an antigen-independent manner, i.e., the interaction frequency and protrusion density remain unchanged during surveillance and immune synapse formation (2). Upon TCR ligation, however, the ensuing antigen-specific interactions appear to lead to a longer dwell time of the microvilli tip in the synaptic area (2) and may even deform the target cell membrane, forming invadosome-like structures (4). It still remains to be investigated whether microvilli and invadosome-like structures are in fact morphologically and functionally similar protrusions. Recent studies from the Husson as well as the Hivroz group (71–73) have revealed the formation of large membrane protrusions after synapse formation, the physiological role of which remains unknown, but may not be confused with microvillar protrusions during diapedesis and immune surveillance.

Microvilli were described as continuously forming under the leading edge of the lamellipodium of migrating T cells. Upon antigen encounter and synapse formation, membrane protrusions can be preferentially observed forming at the synaptic periphery of the T cell–APC interface (4). Importantly, the transient interactions scanning microvilli form with their target surface do not cease after antigen-dependent triggering of the T cell (2), much in line with the findings that synapse maintenance depends on continuous recruitment of new TCR–pMHC interactions (14, 17, 74, 75).

Monitoring and characterizing of T cell microvilli during antigen scanning remains a big challenge within the otherwise extensively researched field of T-cell activation. Previous investigations are confounded by varying cell types, T-cell receptors, and presented ligands, but ultimately by the applied techniques and stimulation platforms. Studies were especially hindered by the lack of methods to investigate three-dimensional and highly dynamic nanostructures on living cells (7). A few research groups applied indirect observation methods to prove the existence of membrane protrusions within the immunological synapse, such as confocal microscopy (4), total internal reflection microscopy (TIRF) (5, 70, 76), and super-resolution techniques (3, 77). Other techniques like lattice light-sheet microscopy and synaptic contact mapping allow a more direct assessment of the membrane structure of activating T cells (2).

### Distribution of Signaling Molecules on Microvillar Protrusions

As extensively reviewed by Orbach and Su (78), microvilli are well-equipped for antigen recognition, and recent studies show that microvillar protrusions are indeed the antigen-sensing entities driving immune surveillance (79–81). In short, TCRs and CD4 coreceptors, CD2 adhesion molecules and essential proteins for T-cell activation, such as Lck and LAT, have been found enriched in microvilli (80). Jung et al. investigated the impact of membrane ultrastructure on TCR distribution on T cells and observed TCR pre-clustering on resting T cells (3). Interestingly, no TCR clusters were observed when T cells were allowed to flatten out on non-activating surfaces (82). Recently, the Ley group observed CD45 exclusion from microvilli tips before antigen recognition (83), whereas Razvag et al. observed an exclusion of CD45 shortly after contact formation (77). Considering the kinetic segregation model, which postulates that T-cell activation is induced by the spatial separation of phosphatases such as CD45 from the phosphorylation sites of TCRs, this implies a high sensitivity of tip-resident TCRs toward antigenic pMHC. Indeed, contacts between microvilli and stimulating surfaces were found to be sufficient for T-cell activation (76, 84). What drives the organization of signaling molecules in microvilli has so far remained elusive, but it is speculated that the extreme membrane curvature and the lipid composition, in particular cholesterol content, may play a role (78). Furthermore, it is generally accepted that microvillar protrusions contain parallel bundles of actin filaments (2, 65) and may colocalize often, but not necessarily always, with TCR molecules [(2) and reviewed in (78)].

In summary, it is evident that microvilli form a restricted reaction volume harboring the necessary molecules for T-cell activation. An exciting observation was recently made by Klotzsch and colleagues, who demonstrated the ability of the T cell to reach into narrowly confined spaces and who showcased the very dynamic nature of their microvillar protrusions and their ability to scan for occluded antigens (85). Interestingly, when T cells reached into micropits below 200 nm in diameter, a slight yet quantifiable antigen-independent cytokine upregulation started. This observation indicates that the signaling molecules within the limited reaction volume of microvilli may be sufficient for cell-body independent signal amplification or, alternatively, that T cells may reach an even higher degree of antigen sensitivity when the dimensions of their microvilli are severely restricted. Effective T-cell activation may hence be aided by enforcing the spatial proximity of signaling molecules downstream of the TCR.

### Microvilli Scanning Speed on Artificial and Natural APCs

The fractal arrangement of microvilli enables T cells to efficiently scan the surfaces of antigen-presenting cells (2). Scanning human CD4^+^ T cells move with a mean velocity of ~3 µm/min over cell surfaces (4). T cells perform immune surveillance in lymph nodes in the presence of antigen with a scanning speed of 2.6 to 5.4 µm/min in a random walk fashion (86, 87), and a dendritic cell typically interacts for ~3 min with individual T cells (88). In resting murine OT-1 TCR-transgenic T cells, microvilli were found to move at an average speed of 5.2 ± 0.4 μm/min, resulting in a 98% coverage of the T cell surface within 1 min. Similarly, the leading edge of the lamellipodium of migrating fibroblasts moves at a velocity of 6.3 µm/min (89). Note that comparable migration velocities have been observed for TCR microclusters in Jurkat T cells (8.4 ± 0.36 μm/min) (90). The mean velocity of actin retrograde flow in Jurkat T cells stimulated with a strong agonist has been recorded to be ~4.8–5.4 µm/min (91, 92), with faster velocities observed for weaker agonists (92).

Motion of any biological membrane in a perpendicular direction to the plane of the receptor–ligand interaction occurs at high frequencies and in the range of tens of nanometers due to thermal fluctuations or stochastic membrane displacement (reviewed in (34)). Perpendicular fluctuations of microvilli tips of about 67 nm were observed within 1 second-long observation windows (70). The lateral movement of microvilli within this experimental setup across a non-activating surface was however minimal, and only rarely spurts of tenths of a micrometer could be observed (70). These results indicate that microvilli move perpendicularly toward a cell surface and retract without much lateral movement to reappear at a different position to continue probing the APC surface. This observation of dynamic microvilli behavior is intriguing, considering that T cells must overcome the glycocalyx in order to probe for surface receptors. Lateral movement through the dense surface layer may be energetically disadvantageous. Also, the geometry of the antigen-presenting surface is likely to influence scanning membrane protrusions (4, 93).

In 2012, Sage et al. performed a series of experiments aimed at determining the depth and width of “invadosome-like” podosomes (ILPs). Although the depth of these structures was found to be stimulus-independent, the width showed a significant decrease in the presence of a specific antigen (4). The authors also showed that calcium flux is initiated ~25 s after the first appearance of an ILP. Interestingly, the presence of antigen caused a substantial stabilization of the ILP lifetime (4), i.e., the transient nature of the scanning ILP ceased to exist after contact with cognate antigen. Cai et al. observed rapid scanning of the opposing surface by microvillar structures and their subsequent arrest or stabilization upon encountering cognate antigen (2). This process seemed, however, to be independent of downstream signaling. A theoretical model has been proposed, attributing the microvilli contact stabilization to the formation of catch bonds (non-covalent bonds whose lifetime increases under force) between TCR and MHC loaded with stimulatory peptide (21), implying a critical role of mechanical forces exerted *via* microvilli in antigen discrimination. The suggested mechanism is in agreement with the finding that microvilli stabilization is independent of actin (2). Further theoretical work indicated that the antigen-dependent arrest of microvilli may indeed be essential for ligand discrimination (94): Within their framework, which was based on kinetic segregation, Fernandes and coworkers found specific TCR triggering if (i) close contact areas between T cells and APCs persisted for at least two seconds and (ii) the radius of the area was smaller than 220 nm.

Considering the mobility of pMHCs on APCs as an additional parameter for T-cell recognition, the field has not yet reached a consensus on to what extent the laterally immobile or the mobile fraction of pMHCs lead to efficient triggering *in vivo* (95–97). Several studies have identified the velocity and quantified the mobile fraction within the membranes of APC (98–100), but to our knowledge, none has shown the functional connection to T cell activation of either fraction. From our own experiments using activating adhesion-competent gel-phase and fluid-phase glass-supported lipid bilayers, we do know that T cells scan and activate efficiently on both surfaces (101), but we could observe a slight delay in response on laterally immobile surfaces, most likely due to a stalled microcluster formation. These results were corroborated by other studies (102, 103). A faster moving pMHC molecule would increase the likelihood of encountering the microvilli tips, which becomes important when contemplating scenarios with very low densities of cognate antigens (21). On the other hand, slower moving or immobilized pMHCs may facilitate rebinding events if microvilli tips stay in close proximity (21), which could influence antigen recognition thresholds (104).

### Force Profile of Membrane Protrusions

As motile entities, T cells experience considerable strain. T-cell protrusions share many properties with filopodia, which are actively used by other motile cells when they screen the surroundings for biochemical and mechanical cues through environment-sensing receptors residing on their tips (105). Filopodia generation requires 5–30 pN (piconewton) of protrusion force (106). Once formed and anchored to cortical actin, they can exert extensive pulling forces on their own (79, 107). Podosome protrusion force was quantified using monocyte-derived cells spreading on a deformable artificial membrane: an average of 29 to 155 nN pushing force was measured, which was positively correlated with substrate rigidity (108). Interestingly, podosomes could adjust their core elasticity toward the substrate rigidity, maintaining a constant indentation depth. Similar results were obtained for podosomes of fibroblasts pushing against SLBs (109). The latter study quantified the molecular tension exerted by individual integrin molecules using DNA-based tension-gauge tethers, hence confirming the tendency of podosomes to predominantly exert perpendicular forces.

*Via* 3D traction force microscopy employing beads bound to an elastic hydrogel, forces of up to several hundred piconewtons exerted by single microvilli were observed (79). Force application *via* membrane protrusions was found to be crucial for the cytotoxic activity of CD8^+^ T cells (110): Knocking out the cytoskeletal regulator WASP not only led to diminished forces and deformation of target cells, but also to a 50% reduction in killing efficiency at low antigen levels.

## Mechanical Forces in T-cell Antigen Recognition

For decades, scientists have attempted to tackle the mystery surrounding the high sensitivity and specificity of T cells for their cognate antigen and have naturally created a multitude of T-cell activation models, each with their own merits and weaknesses. These early models mainly investigated biochemical aspects of the TCR–pMHC interaction, such as the multimeric state of the ligands, the required ligand density for activation, or the influence of co-receptor binding on triggering potency. However, it quickly became apparent that all these models could not sufficiently explain the high sensitivity and specificity of the TCR–pMHC interaction. Eventually, a new parameter entered the field. In 2001, the Dustin group observed that different experimental methods led to marked differences in the measured kinetic parameters of the TCR–pMHC interaction. Consequently, it was hypothesized that the k_off_ may increase due to mechanical force in a 2D setting, where settling T cells interact with immobilized ligands on a surface (111). The intuitive explanation for this phenomenon was the involvement of dynamic cellular processes in destabilizing the TCR–pMHC interaction. Indeed, ten years later, Huppa et al. observed a significant difference in 2D and 3D binding kinetics and, additionally, a pronounced increase in TCR–pMHC interaction lifetime upon the destabilization of the cortical cytoskeleton, indicating that mechanical forces impact T-cell antigen recognition and triggering (112).

**Figure 2:**
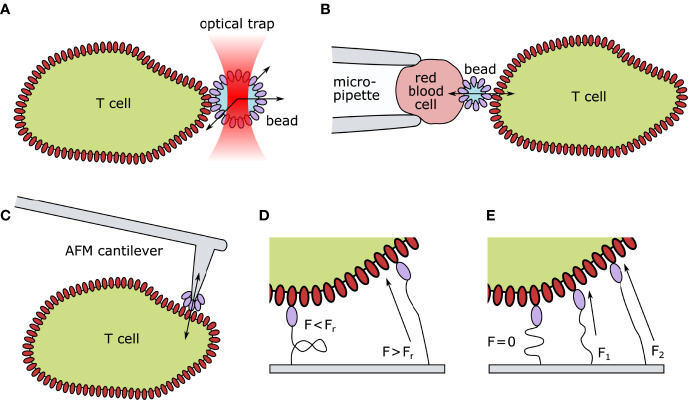
Technical approaches for quantifying mechanical forces exerted on TCR–pMHC pairs. **(A)** Optical tweezer setup: ligand-coated beads are spatially fixed by an optical trap. Upon TCR engagement, the bead is moved out of the laser focus. The deflection indicates the TCR-imposed mechanical force. **(B)** Biomembrane Force Probes: A T cell and a red blood cell are aspirated and held in place *via* a micropipette setup. A ligand-coated bead is attached to the surface of the red blood cells. Upon T-cell contact, altered thermal fluctuation of the bead indicates TCR–ligand engagement. Forces can be exerted by retracting the micropipette. **(C)** Atomic Force Microscopy: A ligand-coated cantilever tip is brought into close proximity of the T cell surface. Upon TCR engagement, the deflection of the cantilever indicates force generation. **(D)** Digital Molecular Force Sensors (MFS): A ligand is attached to a fluorescently labeled MFS unit. In their folded state, the fluorescence is entirely quenched. Such sensors can withstand a certain threshold of strain before (*F_r_*) unfolding. Upon TCR engagement and force generation, the digital MFS unfolds, reducing the quencher efficiency and leading to a quantifiable increase in fluorescence. **(E)** Analog MFS: A ligand is attached to a fluorescently labeled spring unit framed with a FRET (Förster resonance energy transfer) pair. In its coiled state (*F*=0) the fluorophores are in close proximity and the FRET efficiency is high. Upon TCR engagement and force generation (*F*_1_<*F*_2_) the spring unit uncoils continuously decreasing the FRET efficiency between the two fluorophores. AFM, Atomic Force Microscopy; F, Force; F_r_, hairpin rupture force.

The advancement of new techniques followed, with the purpose of identifying the impact of mechanical forces on/during T-cell activation (see **Figure 2** for an overview of the most prominent methods). For this review, we will discriminate between the effects of externally applied mechanical forces and forces exerted through the TCR itself.

### Impact of Externally Applied Mechanical Forces on T-Cell Activation

In a multitude of studies, mechanical forces were applied to the TCR–pMHC bond to characterize the TCR as a mechanosensor. Different approaches were devised to stretch the bond in a defined manner (see overview in **Table 1**):

**Table 1:** Overview of published articles investigating the impact of mechanical forces on T-cell activation.

Forces Activate T cells	Force Amplitude & Direction	# Ligands	Triggered Cells, Stimulus	Ref.
Optical Tweezers	Shear force (50 pN) activates T cells	~10/bead	T cells (murine), pMHCI	(113)
Flow Chamber/Micropipette	Shear/Pulling forces activate T cells	n.d. (cell surface)	T cells (murine), αCD3 on aAPCs	(114)
Atomic Force Microscopy	~20 +/−10 pN/bond sensitivity ~10 pN	1/interface	CD8^+^ T cells (murine), pMHCI	(115)
Micropipette Assay	Applied forces activate T cells	15–30/μm²	CD8^+^ T cells (murine), pMHCI	(116)
Biomembrane Force Probe	Up to ~10 pN/bond enhances lifetime (catch)	1/interface	CD8^+^ T cells (murine), pMHCI	(117)
Optical Tweezer	Up to ~15 pN/bond enhances lifetime (catch)	1/interface	CD8^+^ T cells (murine), pMHCI	(118)
Biomembrane Force Probe	Up to ~10 pN/bond enhances lifetime (catch)	1/interface	CD4^+^ T cells (murine), pMHCII	(119)
Biomembrane Force Probe	Up to ~10 pN/bond enhances lifetime (catch)	1/interface	Pre-pMHC/TCR (murine), pMHCI	(120)
Biomembrane Force Probe	Up to ~10 pN/bond enhances lifetime (catch)	1/interface	CD8^+^ Native/Recombinant TCR	(121)
Optical Tweezer	10–20 pN/bond (in shear & normal direction); Shear forces activate T cells more efficiently	1/interface to 200/interface (20,000 in experiments without force)	CD8^+^ T cells (murine), pMHCI	(122)
Biomembrane Force Probe	Up to ~15 pN/bond enhances lifetime (catch)	1/interface	CD4^+^ T cells (human), pMHCII	(123)

pN, picoNewton; αCD3, antibody against CD3; aAPC, artificial antigen-presenting cell; n.d., not determined; Ref., reference number.

One of the first experimental strategies was to confront T cells with a bead coated with a defined number of TCR–ligands, followed by targeted force application and simultaneous recording of TCR-downstream signaling. A force of as little as 50 pN applied to the pMHC-coated bead in a tangential but not normal orientation with regard to the T cell surface turned out to be sufficient to activate T cells (113). A subsequent study determined the effect of applied shear and pulling forces on the TCR–pMHC bond using artificial APCs and demonstrated that mechanical forces can activate T cells as well in a cell–cell conjugate (114). Forces of as little as 10 pN were found sufficient to induce signaling when imposed through pMHCs or TCR/CD3-specific antibodies (122). Combined, these studies are consistent with the notion that triggering thresholds are not only defined by the intrinsic biochemical properties of TCRs and pMHC but also by force load and directionality. Interestingly, the latter study revealed that forces applied to no more than 1 molecule at the bead–cell interface could not trigger a robust T-cell response, whereas forces applied over 2 bonds resulted in T-cell activation. Given that the physiological concentration of presented antigen *via* pMHCI is estimated to be around 10–100 per interface (124), and as few as 3–10 bonds are sufficient to trigger cytotoxicity (125), these insights let mechanical forces in the context of scanning microvilli tips shine in a new light.

Adding to the multitude of unique insights, the study by Feng et al. provided the first clear evidence that forces applied over multiple bonds at the bead–cell interface are being distributed (122). A prerequisite to load distribution would be the physical coupling of the involved surface area. Along this line of reasoning, the biophysical parameters of membrane curvature and tension recently emerged as a possible mechanism influencing T-cell motility, protrusion, and immune synapse formation (reviewed in (126)). The decisive physical parameter in this model is the modulation of membrane tension and tension decay at the triggering point, which could even be used to explain certain aspects of ligand discrimination (127) and synapse breaking (128).

By measuring TCR–pMHC dissociation kinetics under load with the use of a biomembrane force probe (BFP), Zhu and colleagues found a correlation between the bond lifetime and stimulatory potency (116, 117, 119, 129). BFP probes were used to apply mechanical forces to CD8^+^ (117) and CD4^+^ T cells (119, 123), and showed that for agonistic antigens, forces up to 10 pN prolong the interaction lifetime, forming ‘catch bonds,’ while non-stimulatory pMHCs give rise to much reduced lifetimes under load (‘slip bonds’). By using an optical tweezer setup (118), catch-bond characteristics of stimulatory TCR–pMHC interactions were also observed by others. Furthermore, the directionality of externally applied forces probing the TCR–pMHC interaction impacts the antigen sensitivity of the T cell (122). Therefore, one prevalent model in the field describing antigen discrimination concerns the mechanical probing of each TCR–pMHC interaction. The TCR-imposed molecular forces exerted by the structural dynamics of the cellular membrane help in probing the ligand–receptor interaction, and only strong agonists allow the necessary resistance (life-time) to trigger activation (117, 122). It seems, therefore, plausible that linking forces to synaptic lifetime and the stimulatory potency of a given TCR–pMHC pair manifests as a major principle underlying antigen discrimination in the physiological context of T-cell antigen recognition. However, this has been contested by the recent observation that even agonist pMHCs coated on beads and interacting with a TCR-coated surface exhibited a clear slip bond behaviour behavior under defined flow-generated force in an *in vitro* experiment without cells (130).

### Force Amplitude of TCR-Imposed Mechanical Forces

Adhesion-related forces of about 1–2 nN have been reported within cell–cell contacts. However, measured values varied significantly depending on the cell line under investigation (131, 132). Moreover, net adhesion forces recorded between the conjugated cells correlated with the stimulatory potency of the pMHC (132). Several attempts have since been made to assess forces imposed on a truly molecular level (see **Table 2**).

**Table 2:** Overview of published articles investigating mechanical forces exerted by T cells.

T cells Generate Forces	Exerted Force	Triggered Cells	Ref.
Biomembrane Force Probe	Contact force ~5 pN, ~25 pN (pushing), ~ 2 pN/s loading rate (pulling) [stiffness: 50 pN/µm; sensitivity ~10 pN]	CD4^+^ T cells (murine), αCD3	(71)
Micropillars (TFM)	~200 pN/pillar	CD4^+^ T cells (murine)	(133)
Atomic Force Microscopy	~500 pN/cell (push) & ~800 pN/cell (pull)	CD4+ T cells (murine)	(19)
Digital Molecular Force Sensor	12–19 pN/bond [results given in F1/2 values]	CD8^+^ T cells (murine), pMHCI	(134)
Digital Molecular Force Sensor	>4.7 pN/bond [results given in F1/2 values]	CD4^+^ T cells (murine), αCD3	(135)
Digital Molecular Force Sensor	>4.7 pN/bond [results given in F1/2 values]	CD8^+^ T cells (murine), OT-1, pMHCI, αCD3, anti-PD1	(136)
Analog Molecular Force Sensor (single-molecule resolution)	2–6 pN/bond (activating & scanning conditions) [1.5 pN/s loading rate] 2 pN/bond (scanning conditions)	CD4^+^ T cells (murine), αTCR CD4^+^ T cells (murine), pMHCII	(101)
AFM	Up to 1 nN/cell pushing, 2 nN/cell pulling	CD4^+^ T cells (murine), OT-II TCR, pMHC and αCD3 on AFM cantilevers	(19)
AFM	Up to 2.5 nN/cell	CD4^+^ T cells (murine), 5c.c7 TCR, pMHC on lipid bilayer	(76)
Micropipette Force Probe	Up to 0.5 nN/cell	CD4^+^ T cells (human), αCD3, αCD28	(72)

pN, picoNewton; αCD3, antibody against CD3; αTCR, antibody against TCRβ; Ref., reference number.

Husson et al. (71) adapted the BFP technology with single-molecule detection to visualize the temporal response of single T cells to glass beads functionalized with anti-CD3 and covalently attached to the surface of a red blood cell. Force generation was monitored by the elongation of the red blood cell. In this fashion, distinct pulling and pushing phases could be observed in receptor-engaged T cells.

Traction force microscopy (TFM) has been applied to assess forces related to T-cell adhesion and activation. This methodology involves tracking the force-induced displacement of fluorescent beads embedded in a matrix of defined stiffness resulting from cells crawling or spreading thereon. From such studies, it was concluded that T cells adapt their signaling response according to substrate stiffness. TFM provided the net amplitude and directionality of averaged cellular mechanical forces. With the use of elastic pillars coated with pMHC or activating antibodies, Bashour et al. visualized and quantified TCR-imposed forces (133): T cells deflected pillars with approximately 200 pN per pillar in an antigen-dependent manner. However, the exact number of participating molecular bonds could not be determined. A similar TFM experiment conducted with Jurkat T cells revealed that activating surface conditions (anti-CD3 antibodies) produce higher deforming forces than non-activating conditions (56).

Atomic force microscopy (AFM) using pMHC-coated cantilevers confronting the T cell surface showed distinct pushing and pulling phases without further application of external force (19). The net pulling forces of activating T cells were stronger for high affinity antibodies than for pMHC and absent for control antibodies. Similarly, T cells immobilized on AFM cantilevers exhibited both pushing and pulling forces when contacting activating supported lipid bilayers (76).

Intra- and extracellular molecular force sensors (MFSs) are recent technological additions to the field of nano-mechanosensors and can either have an analog (for peptide/PEG-based sensors) or digital readout (for DNA-based sensors). By defining the mechanical force necessary to unzip a DNA hairpin spanned between a fluorescent dye and a quenching gold particle, Salaita and colleagues successfully measured the range of forces exerted by a defined number of TCR. CD8^+^ T cells were shown to unzip pMHC-carrying hairpins of 12 pN but not of 19 pN resistance, and CD4^+^ T cells unzipped hairpins of 4.7 pN resistance (134, 135). In this follow-up study by the same group, force probe-decorated gold particles were anchored to a fluid lipid bilayer (135). Although this system can be considered mobile, the force probe itself was still immobilized on a rigid surface, resulting in a high counter force. Furthermore, the same technique was applied to gain insights into the mechanical sampling of antigenic peptides of varying potency. The authors reported a correlation between tension, potency, and successful TCR triggering (136).

The shortcomings of digital MFSs can be mitigated by using peptide-based analog MFSs. These contain a flexible peptide whose extension is a continuous function of the applied force. Like a macroscopic spring, the higher the pulling force is exerted, the larger the end-to-end distance of the peptide becomes, allowing for direct measurement of the force. This approach has been showcased by the quantification of forces exerted by single integrins by Morimatsu et al. in 2013 (137). Reasonable estimates of single integrin forces were between 2 and 40 pN (AFM rupture forces), and maximal transmitted forces were measured by a digital DNA-based force sensor to be 20–30 pN. However, peptide-based analog force sensors reported only 1–5 pN (extrapolated from bulk measurements) (137) and 1–3 pN (determined by single-molecule FRET measurements) (138) for individual integrins. There is a considerable difference between the results of these studies depending on the acquisition method. Given the more direct method of data acquisition with single-molecule resolution, the latter studies likely approach the ground truth.

Using an analog peptide-based MFS, we recently quantified the mechanical forces exerted by single TCRs and found a striking difference between activating and scanning conditions (101). We developed a quantitative FRET-based force sensor for application within the immunological synapse, which operates at the single-molecule level (101). As a spring element, we employed a peptide derived from the flagelliform spider silk protein with known elastic properties (139, 140). The biotinylated spring peptide was conjugated to either (i) a stimulating single-chain antibody fragment derived from the TCRβ-reactive H57 monoclonal antibody or (ii) the natural ligand, an MCC-presenting MHC protein. The sensor fits well within the immunological synapse, as it only spans 8 nm in its collapsed configuration (2, 141). We observed 5 to 8 pN force-peaks per TCR for T cells engaging gel-phase and ~2 pN for fluid-phase glass-supported lipid bilayers using the H57-derived MFS, and T cells stimulated with the natural ligand exerted an average force of ~2 pN on gel-phase surfaces. The functional consequences of mechanical forces along the bond axis remain to be investigated, but this study suggests that perpendicular endogenous forces seem negligible. Additionally, temporal single-molecule force profiles revealed a strong molecular force peak of 7.5 pN in early contact situations while T cells scan for antigen and a late arising force peak of 5.6 pN after T-cell activation (101). These results clearly show that force profiles experienced by individual TCR–ligand pairs differ during antigen surveillance and synapse formation. The analog MFS further enabled us to record the time course of force application. We observed linearly increasing forces at a rate of 1.5 pN/s for 2–3 s, followed by a sudden drop to zero, which we speculate is due to TCRs losing their frictional coupling to the actin cytoskeleton.

### Force Orientation During TCR Triggering

The impact of the orientation of the force vector on mechanotransduction is another hotly debated topic in the immune surveillance and T-cell triggering fields. The aforementioned experiments using optical tweezers to pull a bead tangentially or perpendicularly with respect to the T cell surface (113) yielded the first insights into a differential response toward the directionality of externally applied forces. Here, non-agonistic antibodies binding to the CD3ϵγ subunit of the TCR could be used for triggering if pulled tangentially, but not normally with respect to the cell surface. A recent study by the Salaita group yielded the latest insights into the force vector directionality for triggering of TCRs by applying a newly developed technique named SIM-MFM, in which polarization-modulated structured illumination is combined with DNA-based membrane force sensor technology (142). Using an activating antibody against CD3ϵ, they found no preferred direction of TCR-imposed forces. Although these results do not answer the question of force orientation during immune surveillance, the reported method provides a first step toward resolving the force direction required for TCR triggering.

### Impact of Mechanical Forces After Immunological Synapse Formation

Investigating mechanical forces during T-cell activation lays bare numerous differences in the mechanisms employed by various T cell lineages and subtypes. Adhesion cascades, cytoskeletal rearrangement, and, consequently, synapse shape and dynamics are very much dependent on the encountered cellular target. LFA-1 conformation and function clearly depend on the retrograde flow of the actin cytoskeleton and impacts T-cell activation *via* co-stimulation (143, 144). Naïve T cells scanning DCs within the lymphoid tissue experience a rapid LFA-1 maturation cascade in view of the ligand rigidification on the DC membrane (53, 98). The latter study showed that for a DC subset, MHC mobility remained unchanged upon DC cell differentiation (98). Whether MHC molecules also experience mobility changes during or as a result of certain signaling events or synapse formation remains to be addressed (143). Another prominent example of how mechanical forces act during T-cell activation concerns target cell killing by CD8^+^ T cells. As shown by Huse and colleagues, antigen-experienced cytolytic T cells massively strain and deform the target cell membrane surface and promote in this fashion perforin function (145).

## Concluding Remarks

T cells feature specialized membrane protrusions in varying environmental contexts. In the last few years, membrane protrusions have received much attention for their active role during immune surveillance. Microvilli provide the platform for exerting forces based on their cytoskeletal core and their dynamic nature. So far, the community has gained the following insights into the topic:

(i) Microvilli are important for fast and efficient antigen scanning and sampling.
(ii) Microvilli carry all molecules necessary for adhesion and initiating TCR-proximal signaling.
(iii) Microvilli form limited reaction volumes, which may sensitize T cells for antigen.
(iv) Microvilli may not only be involved in the biochemical probing of the environment but may also be necessary for testing the biomechanical properties of the target cells and tissues.
(v) Glycocalyx and cellular stiffness parameters affect TCR triggering.
Considering the dynamic cellular processes surrounding mechanosurveillance, a precise temporal control of the triggering event is a *sine qua non* for further insights in the field, and based on the aforementioned studies, a number of conclusions can already be drawn:
(i) Tensile forces do affect early T cell activation.
(ii) Force transduction is TCR- and peptide-dependent.
(iii) Shear forces or torque affect T cell activation more strongly than normal forces.
(iv) Generated tensile forces are invariably tied to physiological T cell recognition and precede T cell activation.
(v) Individual molecules at protrusion tips are subject to pulling and pushing forces in the pN range.
(vi) Tensile forces are generated internally by rearrangements of the actin cytoskeleton and do not involve the action of acto-myosin motor proteins.
(vii) Immune synapse formation generates mechanical forces that activate ligand-bound integrins.
(viii) T cell-imposed forces deform target cells and promote their killing.

## Open Questions

(i) Are there any lateral movements of antigen-bound microvilli tips? What resistance does the glycocalyx pose to the lateral movement of microvilli? Can the microvillar scanning process be imagined more like a “dragging through the waves” or more like a repeated “poking through the barrier”?
(ii) There is conflicting evidence for increased microvilli life- or dwell times upon antigen encounter. Does antigen binding lead to an arrest of microvilli dynamics? Are such arrests only happening for triggering interactions or also for probing events?
(iii) Is the glycocalyx posing a resistance to microvilli penetration?
(iv) At what point is the force vector reversed from pushing to pulling? How are adhesive interactions influencing this process? Is this process used for ligand discrimination by mechanical probing?
(v) What is the fate of microvillar protrusions after TCR triggering? Are the protein platforms at the microvillar tips evolving to microclusters? What is the impact of LFA-1 maturation on signaling within microvillar protrusions?
(vi) Is the mobility of the pMHCs decisive for the interaction probability and/or rebinding in the context of microvillar scanning? Are target cells mechanically altering their presenting surfaces to allow or inhibit efficient scanning of the surface?
(vii) How are microvilli recognition events coupled to the movement of the entire cell body? How are these signals communicated within the T cell?

## Author Contributions

JG conceived and drafted the manuscript. JG and LS conducted literature review and wrote the manuscript. LS designed the figures. JG, LS, GS, and JH revised the manuscript. All authors listed have made a substantial, direct, and intellectual contribution to the work and approved it for publication.

## Funding

This work was supported by the Austrian Science Fund (FWF) project P32307-B (JG, LS, and GS), and I5056-B (LS and GS), and by the Vienna Science and Technology Fund (WWTF) project LS13-030 (JG, LS, JH, and GS).

## Conflict of Interest

The authors declare that the research was conducted in the absence of any commercial or financial relationships that could be construed as a potential conflict of interest.

## Publisher’s Note

All claims expressed in this article are solely those of the authors and do not necessarily represent those of their affiliated organizations, or those of the publisher, the editors and the reviewers. Any product that may be evaluated in this article, or claim that may be made by its manufacturer, is not guaranteed or endorsed by the publisher.
